# Therapeutic potential of purpurin, a natural anthraquinone dye, in neuroprotection and neurological disorders

**DOI:** 10.1007/s10787-025-01977-7

**Published:** 2025-10-04

**Authors:** Aya M. Mustafa, Ghadir A. Sayed, Shymaa Hatem, Roxane Abdel-Gawad Moussa, Dina M. Hal, Mahmoud A. Mansour, Mohamed S. Abd El Hafeez

**Affiliations:** 1 Department of Pharmacology and Toxicology, Faculty of Pharmacy, Egyptian Russian University, Badr City, 11829 Cairo Egypt; 2 Biochemistry Department, Faculty of Pharmacy, Egyptian Russian University in Cairo, Badr City, 11829 Cairo Egypt; 3 Department of Pharmaceutics and Pharmaceutical Technology, Faculty of Pharmacy, Future University in Egypt, Cairo, Egypt; 4 Department of Pharmaceutics and Industrial Pharmacy, Faculty of Pharmacy, Ain Shams University, Cairo, Egypt; 5 Department of Pharmacognosy, Faculty of Pharmacy, Suez Canal University, Ismailia, 41522 Egypt; 6 Department of Pharmacognosy, Faculty of Pharmacy, Egyptian Russian University, Cairo-Suez Road, Badr City, 11829 Cairo Egypt; 7 Department of Pharmacognosy, College of Pharmacy, University of Kut, Al Kut, Wasit 52001 Iraq

**Keywords:** Purpurin, Anthraquinones, Neuroprotection, Alzheimer’s disease, Bioavailability

## Abstract

Purpurin (1,2,4-trihydroxy-9,10-anthraquinone) is a naturally occurring anthraquinone pigment derived primarily from Rubia species. Beyond its traditional use as a natural dye, purpurin has recently gained attention for its multifaceted pharmacological activities, particularly in neuroprotection. This review outlines the biosynthetic pathways leading to anthraquinone formation via the shikimate, mevalonate, and methylerythritol phosphate routes, culminating in the generation of purpurin. Structure–activity relationship analyses highlight the critical role of hydroxyl substitutions in modulating antioxidant, anticancer, antibacterial, and neuroprotective properties through radical stabilization, DNA intercalation, and metal chelation. Evidence from preclinical studies indicates that purpurin exerts beneficial effects in Alzheimer’s disease, depression, ischemic stroke, and age-related cognitive decline, primarily through anti-tau aggregation, cholinesterase inhibition, serotonergic modulation, antioxidant activity, and anti-inflammatory mechanisms. While purpurin exhibits low acute toxicity and promising pharmacodynamics, its therapeutic translation remains limited by poor solubility, rapid metabolism, and low brain bioavailability. Nanotechnology-based formulations and molecular modifications are being explored to overcome these challenges. Collectively, purpurin represents a versatile bioactive scaffold with considerable potential for the development of multifunctional neuroprotective agents.

## Introduction

Natural products have historically provided a rich source of pharmacologically active compounds (Mustafa et al. 2025), many of which serve as templates for modern drug discovery (Abd Elhafeez 2024). Among these, anthraquinones represent a structurally diverse class of aromatic polyketides widely distributed in plants, fungi, and lichens (Fouillaud et al. 2016). They exhibit a broad spectrum of biological activities, including antimicrobial, anticancer, and neuroprotective effects (Diaz-Munoz et al. 2018).

Purpurin (1,2,4-trihydroxy-9,10-anthraquinone), predominantly isolated from Rubia species such as *Rubia cordifolia* and *Rubia tinctorum*, has long been used as a natural dye due to its intense red coloration (Wen et al. 2022). In recent decades, however, it has drawn increasing interest from the biomedical community for its therapeutic potential (Maria Siril 2023). Its hydroxyl-substituted anthraquinone nucleus confers strong antioxidant capacity, metal-chelating activity, and the ability to intercalate with DNA and interact with key enzymes, thereby underpinning its multifaceted pharmacological actions (Wen et al. 2022), Purpurin, a compound from *Rubia cordifolia*, reduces inflammation and modulates immune responses, showing protective effects against joint damage in rheumatoid arthritis models. It lowers pro-inflammatory cytokines, inhibits MMP3, and is non-toxic (Zeng et al. 2023).

Emerging evidence suggests that purpurin may serve as a promising neuroprotective agent. Preclinical studies demonstrate efficacy in models of Alzheimer’s disease, depression, ischemic stroke, and age-related cognitive decline, largely through mechanisms involving anti-tau aggregation, inhibition of monoamine oxidase-A (MAO-A), reduction of neuroinflammation, and regulation of oxidative stress pathways (Rustage et al. 2025). Despite these encouraging findings, purpurin’s therapeutic translation is hampered by poor aqueous solubility, low systemic bioavailability, and limited brain penetration (Jain 2023).

This review contributes to current knowledge on the biosynthesis of purpurin, its structure–activity relationships, pharmacological properties, distribution, toxicity, and neuroprotective potential. We also highlight recent advances in delivery strategies and future research directions aimed at enhancing its therapeutic utility.

## Purpurin biosynthesis

The biosynthesis of anthraquinones in plants through the shikimic acid (SA) pathway necessitates the involvement of various metabolic systems, including the tricarboxylic acid (TCA), the mevalonate (MVA), and the methyl erythritol phosphate (MEP) pathways (Kang et al. 2022, 2020). The metabolic pathway is predominantly divided into three distinct modules, with the initial module focused on the synthesis of 1,4-dihydroxy-2-naphthoic acid (DHNA). The production of DHNA occurs through a sequence of consecutive enzymatic reactions within the shikimate pathway (Santos-Sánchez et al. 2019). Initially, phosphoenolpyruvate (PEP) and erythrose‐4‐phosphate (E4P) act as substrates in the aldol condensation reaction catalyzed by 3‐deoxy‐7‐phosphoheptulonate synthase (DAHPS), yielding 3‐deoxy‐D‐arabino‐hetulosonate‐7‐phosphate (DAHP). Subsequently, DAHP undergoes dephosphorylation and cyclization mediated by 3-dehydroquinate synthase (DHQS), resulting in the formation of 3‐dehydroquinate (DHQ), which represents the rate-limiting step of the tricarboxylic acid (TCA) cycle. DHQ is subjected to dehydration through the catalytic action of 3-dehydroquinate dehydratase (DHQD), leading to the production of 3‐dehydroshikimic acid (DHS). Salicylic acid (SA) and nicotinamide adenine dinucleotide phosphate (NADP+) are synthesized on the account of shikimate dehydrogenase (SDH) activity. SA engages with adenosine triphosphate (ATP) via the enzymatic action of shikimate kinase to generate shikimic acid-3-phosphate (S3P). The condensation reaction, catalyzed by S3P and PEP and facilitated by 3‐phosphoshikimate 1‐carboxyvinyltransferase (EPSPS), results in the formation of 5‐enolpyruvylshikimate‐3‐phosphate (EPSP). EPSP is subsequently dephosphorylated by chorismate synthase (CS), yielding chorismic acid (CHA). CHA is then converted to isochorismic acid (IC) through the catalytic activity of isochorismate synthase (ICS), which constitutes the rate-limiting step within the shikimate pathway (Dempsey et al. 2011). The O-succinyl benzoate synthase (OSBS) uses the IC and α-ketoglutarate from the TCA cycle as substrates to create O-succinyl benzoate (OSB) and CO2 and PEP. The reaction is catalyzed by thiamine diphosphate (TPP) (Lu et al. 2012). O-succinyl benzoate–CoA ligase (MenE) catalyses the activation of the succinyl side chain of OBS, resulting in the formation of O-succinyl benzoyl–CoA (OSB–CoA). OSB‐CoA is subsequently cyclized to 1,4‐dihydroxy‐2‐naphthoyl‐CoA (DHNA‐CoA) through the enzymatic activity of 1,4‐dihydroxy‐2‐naphthoyl‐CoA synthase (MenB). DHNA‐CoA ultimately produces DHNA by the catalytic action of 1,4‐dihydroxy‐2‐naphthoyl‐CoA thioesterase (DHNAT), which constitutes the A and B rings of the anthraquinone nucleus (Gaid et al. 2012).

In the MVA route, two acetyl-CoA molecules are initially condensed, catalysed by acetyl-CoA carboxylase (ACCA), to produce acetoacetyl-CoA. Acetoacetyl‐CoA and acetyl‐CoA undergo condensation catalysed by hydroxy‐methylglutaryl‐CoA synthase (HMGS) to produce 3‐hydroxyl‐3‐methylglutaryl‐CoA (HMG‐CoA) (Engels et al. 2008). HMG-CoA produces MVA by the enzymatic activity of hydroxymethylglutaryl-CoA reductase (HMGR). MVA utilises one molecule of ATP, catalysed by mevalonate kinase (MVK), to produce mevalonate-5-phosphate (MVA-P), which is further transformed by phosphomevalonate kinase (PMK), consuming another molecule of ATP to yield mevalonate-5-diphosphate (MVADP). MVADP is converted to IPP by the enzyme mevalonate diphosphate decarboxylase. IPP cannot be directly cyclized with DHNA to produce an anthraquinone core structure. It requires catalysis by isopentenyl-diphosphate delta-isomerase (IDI) to produce DMAPP, followed by cyclisation with DHNA.

In the MEP pathway, at first pyruvate reacted with d‐glyceraldehyde‐3‐phosphate to form 1‐deoxy‐d‐xylulose‐5‐phosphate (DXP) under the effect of 1‐deoxy‐d‐xylulose‐5‐phosphate synthase (DXS) (Xiang et al. 2007). Under the reduction isomerization of 1 deoxy d xylulose 5 phosphate reductoisomerase (DXR), DXP needs NADPH to generate MEP. 2‐C‐methyl‐d‐erythritol 4‐phosphate cytidylyltransferase (CMS) produces methylerythritol cytidyl diphosphate (CDP‐ME) with the help of MEP and cytidine 5′‐triphosphate (CTP). Four-diphosphocytidyl-2-Cmethyl-2-d-erythritol kinase (CMK) catalyzes the formation of 4-diphosphocytidyl-2-Cmethyl-2-d-erythritol-2-phosphate (CDP-MEP). 2 C methyl d erythritol 2,4 cyclodiphosphate synthase (MCS) catalyzes CDP-MEP. Two C-methyl-d-erythritol-2,4-cyclodiphosphate (ME-cPP) is the product of cyclization. To create HMBPP, 4‐hydroxy‐3‐methylbut‐2‐enyl-diphosphate synthase (HDS) opens the ring and dehydrates ME‐cPP. 4‐hydroxy‐3‐methylbut‐2‐enyl diphosphate reductase (HDR) catalyzes HMBPP to produce IPP and DMAPP (Wolff et al. 2003; Rohdich et al. 2002).

Condensing steps are covered in the third module. This module combines and cycles DHNA from the SA pathway and DMAPP from the MVA and MEP pathways to create a C ring (Widhalm and Rhodes 2016) and finally form the parent core skeleton of anthraquinone compounds.

**Fig. 1:**
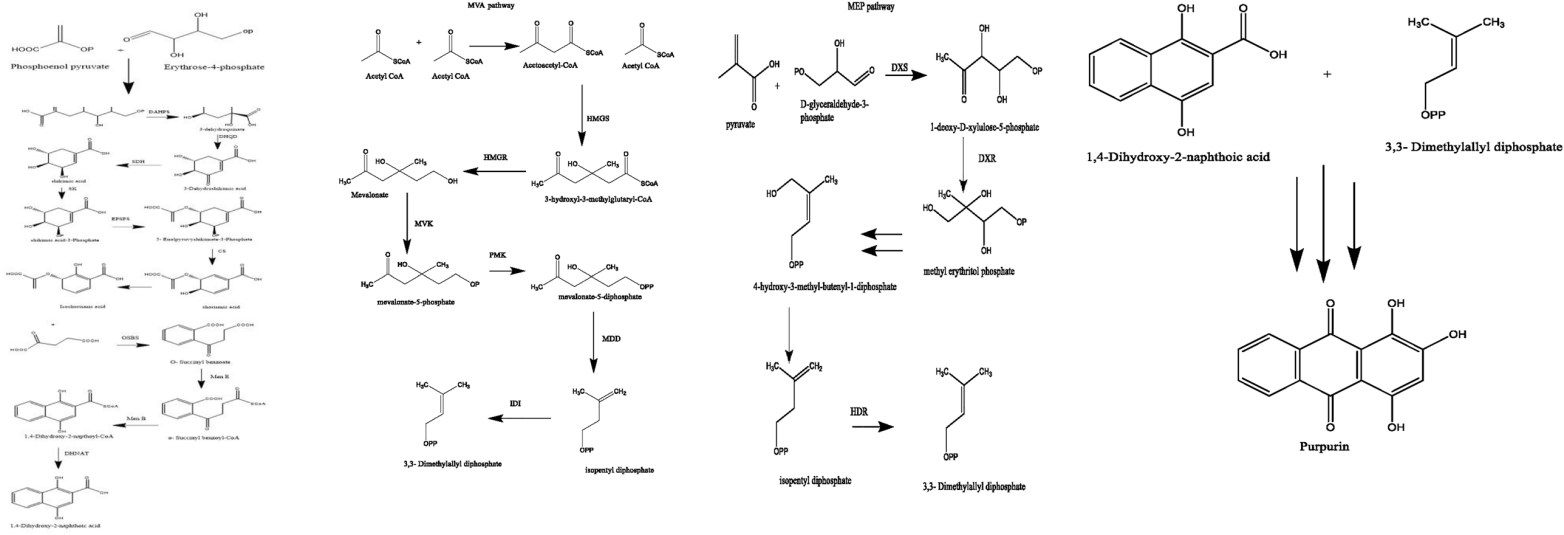
Steps of purpurin synthesis

Hydroxylation steps introduce functional groups at the 1, 2, and 4 positions of the anthraquinone ring to form purpurin (1,2,4-trihydroxy-9,10-anthraquinone). Figure 1 shows the steps for synthesis of purpurin.

## Structure activity relationship of purpurin

Purpurin (1,2,4-trihydroxyanthraquinone) is a naturally occurring anthraquinone pigment predominantly located in the genus *Rubia*. Its bioactivities, including antioxidant, anticancer, antibacterial, and neuroprotective effects, are strongly associated with its anthraquinone nucleus and hydroxyl substitution pattern (Shang and Mohideen 2023). The anthraquinone core offers the planar aromatic framework essential for *π*–*π* stacking with DNA, intercalation, and enzyme interactions. Facilitates redox cycling and regulation of reactive oxygen species (ROS). C-1 Hydroxyl (phenolic OH) is regarded as essential for hydrogen bonding with target biomolecules (e.g., topoisomerases, kinases) and improves antioxidant capabilities by donating hydrogen to free radicals. Furthermore, the presence of C-2 Hydroxyl in an ortho position to C-1 OH facilitates intramolecular hydrogen bonding, which stabilises phenoxyl radicals and enhances antioxidant efficacy. The para-positioned C-4 hydroxyl relative to the C-2 hydroxyl facilitates prolonged conjugation and electron delocalisation, hence improving radical scavenging and chelation with metal ions (Fe^2^⁺, Cu^2^⁺) (Wang et al. 2024a). The antioxidant activity is augmented by various ortho- and para-hydroxyl groups in relation to the quinone carbonyls. The 1,2-OH and 2,4-OH configurations enhance radical stabilisation via resonance. Hydroxyl groups facilitate preferential binding to cancer cell DNA and enzymes such as topoisomerase II, enhancing metal chelation (Fe^2^⁺), which induces Fenton-type reactive oxygen species production and apoptosis. Hydroxyl substitution enhances antibacterial effectiveness via increasing contact with microbial cell wall proteins and DNA. The hydrophobic aromatic core enhances penetration into lipid membranes. The methylation or glycosylation of hydroxyl groups diminishes antioxidant and DNA-binding capacities, although may enhance bioavailability. Halogen substitution on the aromatic ring augments lipophilicity, thereby amplifying antibacterial efficacy while possibly diminishing antioxidant capacity. The incorporation of bulky groups may obstruct DNA intercalation while perhaps enhancing selectivity towards enzymes. Structural comparisons indicate that the 4-hydroxy group is essential for binding affinity and selectivity. Molecular docking demonstrated hydrogen bonding between purpurin and MAO-A (Singh et al. 2021).

## Distribution of purpurin

Purpurin, a key anthraquinone pigment of Rubia species, has been quantified at varying levels depending on species, cultivation conditions, and analytical methodology. In *Rubia cordifolia*, root extracts analyzed by UHPLC-PDA across different Chinese regions revealed concentrations up to 3.03 mg/g, with a mean of 0.303 mg/g (range 0.00–3.03 mg/g) (Wang et al. 2024b). Complementary work on wild accessions of the same species, employing HPTLC densitometry with ISSR population profiling, reported purpurin contents of up to 0.28% *w*/*w* (Natarajan et al. 2019). In *Rubia tinctorum*, in-vitro hairy-root cultures yielded substantially higher levels, reaching 0.594% (5.94 mg/g), as determined by RP-HPLC with diode-array detection and external standards (Bányai et al. 2006).

Broader comparative analyses of *R. peregrina, R. akane, R. sikkimensis*, and *R. yunnanensis* showed that textiles dyed with these species consistently contained purpurin, although quantitative values were not provided; instead, HPLC-PDA/HR-MS was used to establish species-specific anthraquinone profiles (Mouri and Laursen 2012). In addition, *R. tinctorum* was shown to contain purpurin that was successfully isolated and structurally confirmed through HPLC-UV-VIS combined with NMR, though without quantitative estimation (Marković et al. 2013). Collectively, these findings highlight not only the variability in purpurin content across Rubia taxa and growth systems but also the diversity of chromatographic and spectroscopic methods employed in its detection and characterization. Table 1 summarizes the distribution of purpurin.

**Table 1:** Distribution of purpurin

Plant name	Family	Amount	Method used	Ref
*Rubia cordifolia*	Rubiaceae	Up to 3.03 mg/g root (0.303) (Range 0.00–3.03)	UHPLC-PDA analysis of root extracts sampling different Chinese regions	Wang et al. (2024b)
*Rubia cordifolia*	Rubiaceae	Up to 0.28% *w*/*w* in wild accessions	HPTLC densitometry and ISSR population profiling using external standards	Natarajan et al. (2019)
*Rubia tinctorum*	Rubiaceae	Up to 0.594% (5.94 mg/g) in hairy-root cultures	RP-HPLC with external standard, diode-array detection in-vitro culture	Bányai et al. (2006)
*Rubia peregrina, Rubia akane, Rubia sikkimensis, Rubia yunnanensis*	Rubiaceae	Textiles dyed with all the species examined contain varying amounts of purpurin, however quantitative data were not mentioned	HPLC-PDA/HR-MS Profiling of root extracts, identification of anthraquinone patterns	Mouri and Laursen (2012)
*Rubia tinctorum*	Rubiaceae	Purpurin isolated and characterized, though quantitative data were not mentioned	HPLC-UV-VIS with NMR-based confirmation	Marković et al. (2013)

## Neuroprotective effects of purpurin across neurological disorders

**Fig. 2:**
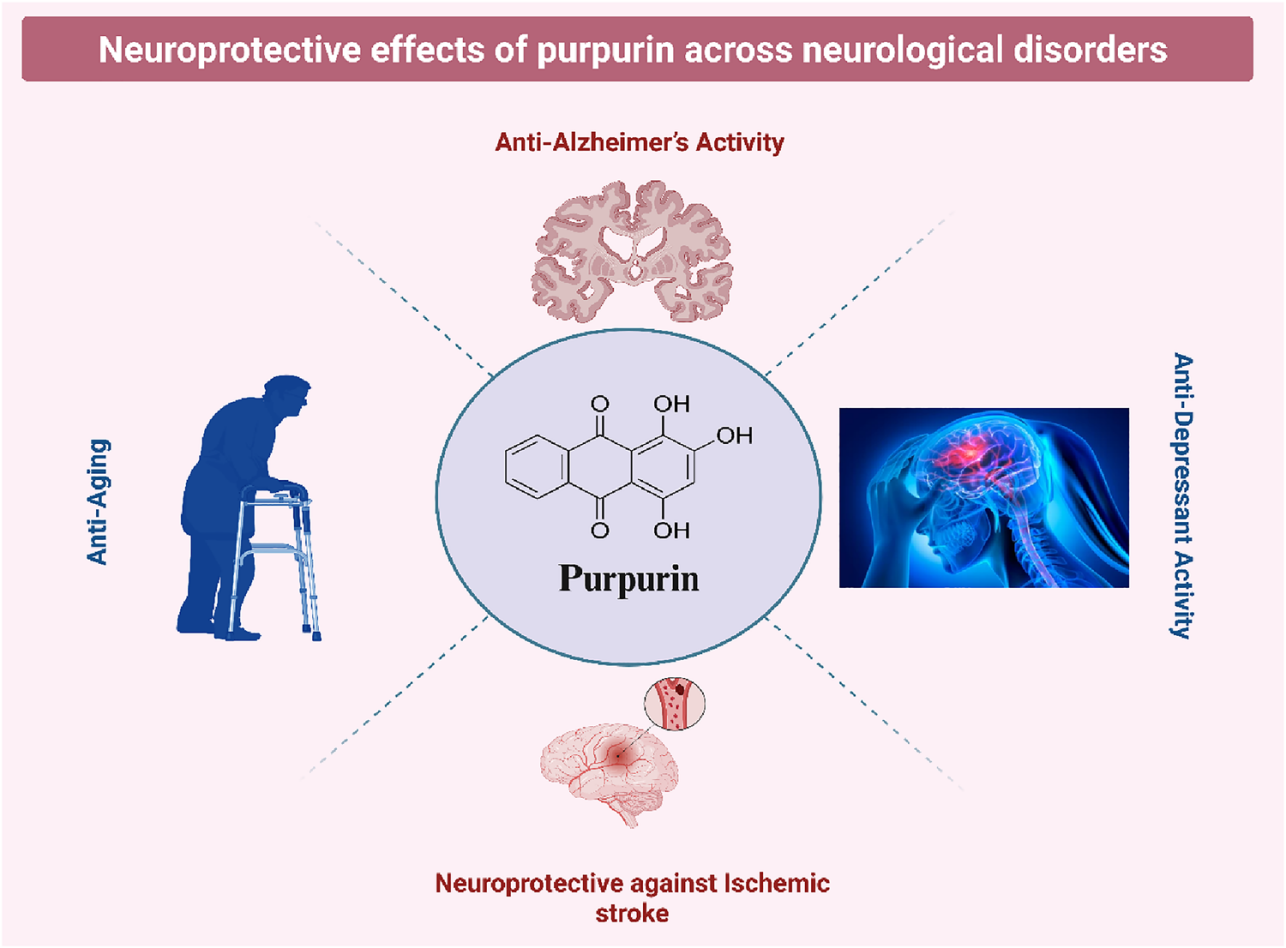
Neuroprotective effects of purpurin across neurological disorders

Purpurin, a naturally occurring anthraquinone, has gained attention for its broad neuroprotective potential across various neurological disorders. It has demonstrated therapeutic promise in models of Alzheimer’s disease, depression, ischemic stroke, and age-related cognitive decline. These findings highlight purpurin as a versatile candidate for the development of novel treatments targeting multiple brain pathologies. Table 2 and Fig. 2 summarize the neuroprotective effects of purpurin across neurological disorders.

**Table 2:** Neuroprotective effects of purpurin across neurological disorders

Condition	Mechanism of action	Experimental models	Key molecular targets/pathways	Key outcomes	References
Alzheimer’s Disease	Anti-tau aggregation, anti-inflammatory, cholinesterase inhibition	Transgenic Drosophila (WT-FL hTau), in-vitro Tau aggregation assays, hTau-overexpressing cells	Tau PHF6 inhibition, ↓ hTau phosphorylation, NLRP3 inflammasome, AChE/BuChE inhibition	↓ tau accumulation, disrupted PHF6 fibrils, ↓ IL-1β, IL-6, TNF-α, strong AChE/BuChE inhibition, BBB permeability	Chen et al. (2020), Singh et al., (2021), Viswanathan et al. (2020), Zengin et al. (2016)
Depression	MAO-A inhibition, serotonergic modulation, 5-HT₁A receptor activation	*p*-chlorophenylalanine-induced model, MAO isoform assays	MAO-A (IC_50_ = 2.5 μM; Ki = 0.422 μM), ↑ 5-HT, 5-HT₁A pathway, ↓ 5-HIAA/5-HT ratio	↓ depressive behaviors, ↑ serotonergic activity, selective MAO-A inhibition, high docking affinity (− 40 kcal/mol)	de Beer et al. (2020), Lee et al. (2017), Ma et al. (2020)
Ischemic Stroke	Antioxidant, anti-inflammatory, anti-apoptotic	MCAO rat model	↑ SOD, CAT, GSH; ↓ MDA, TNF-α, IL-1β, IL-6; ↓ Bax, cleaved caspase-3; ↑ Bcl-2	↓ infarct volume, improved neurological scores, ↓ neuronal apoptosis, stabilized mitochondrial potential	Kim et al. (2022)
Aging	Antioxidant, anti-inflammatory, neurogenesis enhancement	d-galactose-induced aging mouse model, hippocampal cell assays	↓ ROS, MDA, JNK phosphorylation, caspase-3; ↑ Ki67 + , DCX + cells; ↓ IL-1β, IL-6, TNF-α	↑ memory performance, ↑ neurogenesis, ↓ neuroinflammation, ↓ apoptosis	Kwon et al. (2023)

### Alzheimer’s disease

Alzheimer’s disease (AD) is a progressive neurodegenerative disorder marked by pathological features such as tau protein aggregation and hyperphosphorylation, chronic neuroinflammation, and cholinergic dysfunction mediated by acetylcholinesterase (AChE) and butyrylcholinesterase (BuChE). Recent research underscores purpurin, a natural anthraquinone, as a prospective multi-target therapeutic agent in the treatment of Alzheimer's disease (Rustage et al. 2024).

In-vivo investigations utilizing transgenic Drosophila models expressing wild-type full-length human tau (WT-FL hTau), purpurin exhibited considerable neuroprotective effects. These benefits are probably facilitated by its capacity to suppress tau accumulation and diminish tau phosphorylation, thus alleviating tau-mediated neurotoxicity. In a high-throughput screening assay employing a proxy model, purpurin was demonstrated to prevent roughly 50% of aggregation-prone PHF6 hexapeptide fragments of tau, which are significant contributors to paired helical filament (PHF) production. Furthermore, in-vitro experiments demonstrated that purpurin not only inhibited PHF6 aggregation but also dismantled pre-formed fibrils by interacting with PHF6 residues critical for sustaining *β*-sheet conformation (Viswanathan et al. 2020).

Cell-based tests with hTau-overexpressing models that support these results showed that purpurin therapy led to a decrease in intracellular hTau accumulation, which shows that it has a broad anti-tau action. Purpurin has been demonstrated to be able to cross the blood–brain barrier, which makes it more useful for treating diseases of the central nervous system (Singh et al. 2021).

In addition to its anti-tau actions, purpurin has shown promise in reducing neuroinflammation, which is a key part of what causes Alzheimer's disease. It especially lowered the expression of the NLRP3 inflammasome, which is a crucial regulator of how the brain's innate immune system works. Molecular docking studies confirmed these results, showing that purpurin strongly binds to the active region of NLRP3 (docking score =  − 12.9755) and is stabilized by two hydrogen bonds with arginine 165 (Arg165) and threonine 167 (Thr167) (Chen et al. 2020).

Cholinesterase inhibition is another way that purpurin protects the brain, and it is also a key symptomatic treatment for Alzheimer's disease. In-vitro studies have demonstrated that purpurin is a strong inhibitor of AChE (IC_50_ = 10.14 µg/mL) and BuChE (IC_50_ = 7.09 µg/mL). This means that it could boost cholinergic neurotransmission (Zengin et al. 2016).

Taken together, these data showed that purpurin influences multiple important pathways that are involved in the progression of Alzheimer's disease, such as tau pathology, neuroinflammation, and cholinergic dysfunction. Purpurin is a promising candidate for further development as a multifunctional neurotherapeutic agent because it can control various pathways, cross the blood–brain barrier, and has low cytotoxicity.

### Depression

Recent research indicates that purpurin demonstrated significant antidepressant effects via many pathways related to the serotonergic system and the inhibition of monoamine oxidase (MAO) (Lee et al. 2017).

In an in-vivo study, long-term injections of purpurin significantly reduced behaviors that were similar to depression in a model of depression caused by *p*-chlorophenylalanine, which is known to lower serotonin levels in the brain. People thought that purpurin worked as an antidepressant because it increased serotonin activity in the brain and stopped MAO from breaking down serotonin. Through pharmacological interventions, we were able to confirm the role of the 5-HT₁A receptor pathway in its mechanism. The selective 5-HT₁A receptor antagonist WAY-100635 blocked the effects of purpurin, while the 5-HT₁A agonist 8-OH-DPAT and a low dose of the SSRI antidepressant fluoxetine increased its effects (Ma et al. 2020).

Purpurin is a reversible, competitive, and selective inhibitor of monoamine oxidase-A (MAO-A) at the molecular level. MAO-A is an important enzyme that breaks down serotonin and other monoamines in the brain. When tested in-vitro on recombinant human MAO isoforms, purpurin had a strong effect on MAO-A, with an IC_50_ of 2.50 μM and a Ki of 0.422 μM. It had little effect on MAO-B. This selectivity makes it a better antidepressant without affecting MAO-B-related pathways, which are more important for Alzheimer's and Parkinson's diseases (Fowler et al. 2010; Lee et al. 2017). Additionally, structure–activity relationship (SAR) studies have shown that the 4-hydroxy group of purpurin is necessary for its MAO-A inhibition. Molecular docking simulations confirmed these findings by showing that MAO-A binds more strongly (− 40.0 kcal/mol) than MAO-B (− 33.9 kcal/mol). The docking model found Ile207 and Gly443 to be important amino acids that help purpurin form hydrogen bonds in the active site of MAO-A (Lee et al. 2017).

One study identified purpurin as a selective MAO-A inhibitor, however another investigation demonstrated that purpurin inhibited both MAO-A (IC_50_ = 7.99 μM) and MAO-B (IC_50_ = 40.2 μM), with a pronounced selectivity for MAO-A. The inconsistencies may be ascribed to variations in test conditions or extract purity (de Beer et al. 2020).

These results point to purpurin as a potential antidepressant that works by blocking MAO-A, raising serotonin levels, and changing how 5-HT₁A receptors signal. These methods show that purpurin could be a good starting point for making new antidepressant treatments that have fewer side effects compared to existing pharmaceutical drugs.

### Ischemic stroke

Purpurin has shown to be very protective of the brain in models of ischemic brain injury, mostly because it is an antioxidant, anti-inflammatory, and anti-apoptotic agent. Purpurin significantly reduced infarct volume and neurological deficits in a rat model of middle cerebral artery occlusion (MCAO). This protection was connected to its ability to reduce oxidative stress by increasing the activities of endogenous antioxidant enzymes (SOD, CAT, and GSH) and lowering lipid peroxidation markers like MDA. Moreover, purpurin attenuated neuroinflammation by downregulating critical proinflammatory cytokines (TNF-α, IL-1β, IL-6) and inhibiting the activation of microglia and astrocytes. It also reduced neuronal apoptosis by regulating Bcl-2 family proteins, diminishing Bax and cleaved caspase-3 levels, and enhancing Bcl-2 expression. Significantly, purpurin inhibited mitochondrial malfunction and preserved membrane potential, hence reinforcing its involvement in cellular survival after ischemia injury. The findings indicate that purpurin may act as a potential neuroprotective drug in ischemic stroke by modulating oxidative and inflammatory processes through many targets (Kim et al. 2022).

### Aging

Purpurin had strong neuroprotective benefits against brain degeneration that happens with age, mostly because it is an antioxidant and an anti-inflammatory. In a mouse model of aging caused by d-galactose, purpurin significantly enhanced cognitive ability, as shown by better spatial memory in the Morris water maze test. Mechanistically, it reduced oxidative stress by lowering reactive oxygen species and lipid peroxidation, while also restoring the viability of hippocampal cells in-vitro. It also lowered the phosphorylation of c-Jun N-terminal kinase (JNK) and the activation of caspase-3, which are both connected to neuronal apoptosis. In-vivo, purpurin slowed down the age-related reduction in neurogenesis in the hippocampus by increasing Ki67+ proliferative cells and doublecortin (DCX)+ neuroblasts in the dentate gyrus. It also lowered neuroinflammation by lowering the activity of microglia and lowering the levels of pro-inflammatory cytokines such IL-1β, IL-6, and TNF-α in the hippocampus. The presented information supports the idea that purpurin could be a neuroprotective medicine that slows down cognitive loss that happens with age by controlling oxidative and inflammatory processes (Kwon et al. 2023).

## Toxicity of purpurin

Purpurin, a naturally occurring anthraquinone molecule, has undergone comprehensive in-vivo investigations to assess its long-term safety and potential toxicological effects. A chronic toxicity experiment that lasted 520 days showed that long-term oral therapy with purpurin at a dietary level of 1% had serious negative effects. In this experiment, one group of animals was fed a diet containing 1% purified purpurin, while a control group received no purpurin. The results were striking: rats in the purpurin group showed reduced body weight gain, a high incidence of chronic progressive nephropathy (27 out of 27 rats), and notable pathological changes in the urinary bladder, including hyperplasia in 15 animals and carcinomas in 5 animals. Additionally, Leydig cell tumors were detected in 22 of the purpurin-treated rats, indicating a broader systemic carcinogenic influence. Histopathological examinations revealed that the kidney was a primary target of toxicity, with extensive tissue damage, while the bladder showed both pre-neoplastic and neoplastic lesions consistent with tumor promotion. Interestingly, purpurin did not significantly affect the intestinal tract, highlighting variability in tissue susceptibility among hydroxyanthraquinones. Taken together, these findings emphasize that purpurin possesses strong nephrotoxic and carcinogenic properties, particularly in the urinary system, and may act as a promoter of bladder cancer. The study underscores the importance of considering both therapeutic potential and long-term risks of naturally derived compounds, as purpurin exemplifies how environmental agents of natural origin can still pose significant hazards to health and potentially contribute to human carcinogenesis. Histopathological studies of male F344 rats on this treatment plan showed a lot of hyperplasia in the pelvic epithelium and the appearance of neoplastic lesions in the urinary bladder. The lesions comprised benign papilloma and malignant carcinomas, signifying a tumorigenic potential linked to persistent purpurin exposure (Mori et al. 1996).

The presence of prominent crystalline deposits in the renal pelvis and urinary bladder was a notable pathological feature identified in this investigation. It is hypothesized that these crystal forms develop following an initial noxious attack by purpurin on the renal tubular epithelium. This kidney injury may result in chronic irritation and compensatory epithelial hyperplasia, thereby increasing the likelihood of neoplastic transformation. These results underscore the importance of evaluating kidney toxicity and crystal nephropathy in the safety assessment of bioactive substances that are naturally produced (Rustage et al. 2024).

In order to determine whether purpurin's biological effects were associated with cytotoxic pathways, an in-vitro cell viability experiment was implemented using the MTT technique. To assess potential cellular toxicity under sublethal conditions, purpurin concentrations were maintained at or below 50 µM in macrophage cell lines. The results suggested that purpurin had negligible cytotoxic effects, with cell viability primarily maintained across the evaluated dosages. A slight decrease in cell viability of approximately 11% was observed at the maximal measured concentration of 50 µM. This minor reduction does not indicate significant toxicity, suggesting that purpurin does not directly induce cell demise in macrophage cells under these experimental conditions (Nam et al. 2017).

Acute oral toxicity experiments conducted in accordance with OECD 423 guidelines corroborated the comparatively low toxicity profile of purpurin. The oral administration to mice revealed a fatal dosage 50 (LD_50_) exceeding 2000 mg/kg. According to this criterion, purpurin was designated as a Category 5 toxicant, signifying a comparatively low degree of acute oral toxicity under established testing protocols (Bedi and Krishan 2020).

The available data collectively suggests that purpurin is well-tolerated during acute exposure and exhibits minimal cytotoxicity in-vitro. However, protracted treatment at elevated doses may pose a risk of organ-specific toxicity, particularly in the renal and urinary systems. The importance of meticulous long-term safety assessments and dose optimization in the development of purpurin-based therapies or nutraceuticals is emphasized by these findings.

Purpurin is a naturally occurring anthraquinone molecule obtained from the roots of the *Rubia tinctorum* plant, which has traditionally been used as a red dye. In recent decades, it has received attention for its neuroprotective characteristics, notably in the context of conditions like ischemic stroke, Alzheimer's disease, and depression (Rustage et al. 2024). Its medicinal efficacy originates from its powerful anti-inflammatory and antioxidant properties, which serve to reduce oxidative stress and modify signaling pathways such as MAPKs and Bax/Bcl-2 implicated in neuronal apoptosis.

Conversely, many market products used to treat neurological diseases target a specific biochemical pathway and may trigger major side effects such as gastrointestinal and cardiac problems, kidney and liver damage, mood swings and cognitive impairment (Chen et al. 2024).

However, its bioavailability is hindered by factors such as solubility and metabolic decomposition. Purpurin is intensively metabolized in the gastrointestinal system and liver after consumption, significantly reducing its bioavailability (Zhang et al. 2024; Parthasarathy et al. 2025). This implies that just a small portion of purpurin reaches the bloodstream and then the CNS, demonstrating its low concentration in brain (Noor et al. 2024).

Following oral administration of *Rubia tinctorum* extract in rats, purpurin reached an insignificant plasma concentration (*C*_max_ = 70.10 ± 11.78 ng/mL), indicating modest systemic exposure. Poor aqueous solubility, fast degradation by CYP450 enzymes (particularly CYP1A2 and CYP3A4), and potential first-pass hepatic metabolism all contributed to its low bioavailability, cumulatively lowered its therapeutic window (Rustage et al. 2025).

Although it can pass the blood–brain barrier, its retention in neural tissues varies, and its half-life and clearance rates have not been thoroughly studied. Furthermore, most pharmacokinetic data are derived from preclinical models, with no clinical trials reported to date (Zhao et al. 2024).

Accordingly, increasing purpurin's bioavailability in the CNS is critical for boosting its therapeutic potential. Several techniques have been developed to alleviate the challenges associated with its absorption and administration (Zhao et al. 2024). Nanoparticle encapsulation, lipid-based carriers, cyclodextrin complexation, and the implementation of permeability enhancers have all been investigated as strategies for improving brain delivery (Pandey and Kumar 2025).

Recently, purpurin (PUR) has been the focus of research to prove its neuro-modulatory qualities, particularly in depression, Parkinson, and Alzheimer's disease. Several research were carried out to fully understand PUR neuroprotective effects, yet they exerted little progression regarding its formulation, using only the drug as solo component in the treatment strategy without using any targeting moieties, absorption enhancing agents, etc.

Zhao et al. (2024) reported that about 267 natural compounds exhibited close relationships with AD. They found that 19 natural compounds could have a protective impact in at least one AD-related cell model, among them purpurin was the most prominent and effective natural molecule. Purpurin boosted synaptic plasticity and the neurotrophic impact in the brains of rats given d-galactose, decreased oxidative stress damage and neuroinflammation, and improved cognitive learning and memory skills.

The amazing antidepressant-like activity of PUR was followed by (Ma et al. 2020) who conducted a detailed study on experimental rodents. PUR was simply administered as drug solution via gavage. The results proved a significant decrease in immobilization time in both, forced swim test (FST) and tail suspension test (TST), after chronic administration of PUR for over three weeks’ time period It is to be noted that the same doses of PUR that proved to be effective on TST and FST did not affect the locomotor activity in mice, even when administered at the same regimen. The same study also postulated that PUR doesn’t exert any antidepressant activity after acute dosing. PUR impact on the behavioral and neuroendocrine responsiveness consequences of intracerebroventricular (ICV) injection of corticotrophin releasing factor (CRF) was also assessed. Significant neuroendocrine reactivity was induced by this injection, as evidenced by elevated ACTH and CORT levels. Chronic PUR therapy could significantly reduce these ACTH and CORT spikes. Regarding its inhibitory effect on monoamine oxidase activity A and B (MOA), PUR chronic use succeeded in increasing the level of 5-HT and decreased the ratio of 5-HIAA/5-HT in the hippocampus, hypothalamus, and frontal cortex. The mouse brain's elevated 5-HT level and decreased 5-HIAA/5-HT ratio prove that chronic use of PUR can indeed affect monoamine metabolism (Suryawanshi and Rajendra 2023).

PUR neuroprotective effect against Alzheimer’s disease-like symptoms was followed by Kim et al. based on its effect on ischemic damage (Kim et al. 2022). In this study, levels of JNK, ERK, p38 proteins, and their phosphorylated forms were followed; the ratio of p-JNK/JNK, p-ERK/ERK, and p-p-p38/p38 was significantly lower in PUR group than in control group. Moreover, the changes in Bax and Bcl-2 levels were improved significantly by oral PUR administration. The levels of Bax protein rose to 224.4% of the control group, a considerable rise. Bax levels were 142.2% lower in the purpurin-treated group than in the vehicle-treated group, which was a significant decrease.

Similarly, Chen et al. (2020) proved the superiority of Purpurin and Rhein in treating Alzheimer’s disease. This conclusion came out after twelve quinones been investigated using molecular docking technique between quinones and NLRP3 (nucleotide-binding domain leucine-rich repeat (NLR) and pyrin domain containing receptor 3).

Although recent research was focusing on understanding this neuroprotective effect, few works were done to formulate PUR in a novel formulation or delivery system. Unfortunately, the blood–brain barrier prevents many therapeutic compounds from entering the brain, making it difficult to translate PUR encouraging findings into clinical application. Researchers should be using nanotechnology as a solution to this problem, in addition to PUR sluggish absorption following oral administration, the *t*_max_ is reached after approximately 1.61 ± 1.24 h (Rustage et al. 2024). Formulation into NPs is supposed to provide a useful method to increase its absorption, bioavailability, and ability to be delivered precisely to the brain's site of action. Unfortunately, no nanoparticle, liposomal, polymeric, or excipient-based formulation studies exist specifically for purpurin itself, although several research focused on one of its derivatives only known as PUR-18.

PUR-18, a derivative of PUR linked with chlorophyll, was recently the focus of research. This combination was found to have strong photosensitizing effect that could be useful especially in cancer management (Yeo et al. 2022). By deactivating toll-like receptors, Zengin et al. hypothesized that a PUR-18 phytol ester reduced lipopolysaccharide (LPS)-induced NO generation in RAW 264.7 cells by 80% at concentrations of 100 μg/ml and 10–100 μg/ml. PUR reduces intracellular oxidative stress, which in turn controls inflammation (Zengin et al. 2016).

A detailed study was performed by (Boncel et al. 2016), on Fe-encapsulated multiwall carbon nanotubes (Fe@MWCNTs) for enhancing its anticancer activity in breast cancer. They adopted several drugs and techniques and finally got the conclusion that the Fe@MWCNTs that were most cytotoxic and steerable in a magnetic field toward promising formulation loaded with 5-fluorouracil in a *β*-cyclodextrin cage or with covalently linked purpurin.

PUR-18 was also formulated as SLNPs (solid lipid nanoparticles) in order to improve its bioavailability. Yeo et al. (2022)., confirmed the enhancement of the photodynamic therapy (PDT) effect of PUR-18 after inclusion in SLNPs. This could increase its anticancer potential on tumor cells. Worthy to mention that the same research team, few years later, created purpurin-18-N-aminoimide methyl ester (P18 N AI ME) and encapsulate it in lipid nanovesicles (LNVs) aiming to enhance its PDT effect (Yeo et al. 2025; Wassif et al. 2024).

Similarly, Liu et al. (2008) improved the PDT effect of PUR-18 by enclosing it in magnetic silica nano carriers. PEGylation was also a very useful technique (Pavlíčková et al. 2019). When compared to the parental PUR-18, the photosensitizing efficacy was greatly increased by the addition of a zinc ion and PEGylation, which reduced the IC_50_ in HeLa cells by up to 170 times (Pavlíčková et al. 2019).

Being a natural plant extracted quinone molecule, a recent research conducted by (Wijesinghe et al. 2021) made use of its green nature to synthesize zinc oxide (ZnO) NPs without making use of its pharmacologic neuroprotective activity. They adopted T. purpurea leaf, stem, and flower extracts to greenly synthesize ZnO NPs using. The genus Tephrosia contains more than 400 species that are found in tropical areas of the world. Worth mentioning that T. purpurea contains numerous phytochemicals like retinoids, B, glycosides, fatty acids, and several flavonoids including purpurin.

A recent research by Chitgupi et al. (2018) also used PUR in construction PUR-phospholipid system as a drug carrier, without making any use of PUR pharmacologic activity. This carrier is a lipid chromophore that is 30 nm redshifted in near-infrared absorbance when compared to pyropheophorbide-phospholipid (Pyr-P), which is chemically identical. Small quantities of Pur-P or Pyr-P in liposomes showed comparable physical characteristics and fluorescence self-quenching. Pur-P and Pyr-P liposomes were loaded with different cargos, combined into a single colloidal suspension, and their cargos were released selectively based on the wavelength of the irradiation. Controlling the positioning of multicolor lasers allowed for the spatiotemporal control of unique cargo release.

Combination of PUR with other neuroprotective drugs was attracting attention recently, in which (Ma et al. 2020) showed that co-administration of PUR with a 5-HT receptor agonist (8-OH-DPAT), at subclinical doses of each, evoked in a remarkable potentiated antidepressant-like activity. The extensive studies of PUR antidepressant-like activity pointed to the critical role played by the serotonergic system (5-HT), in contrast to nor-adrenaline (NA) system that did not affect PUR activity at all. This could explain the advantage of combining PUR with fluoxetine in the treatment of major depression.

## Conclusion

Purpurin exemplifies a natural compound with a unique combination of structural, pharmacological, and therapeutic attributes. Its diverse biological actions, particularly in mitigating oxidative stress, inflammation, and neurodegenerative processes, position it as a promising candidate for the management of complex neurological disorders. However, long-term safety concerns, renal toxicity at high doses, and poor pharmacokinetic properties pose significant challenges to clinical translation. Advances in drug delivery systems, such as nanoparticle encapsulation and lipid-based carriers, alongside rational structural modifications, may substantially improve its bioavailability and therapeutic index. Future research should prioritize targeted formulation strategies, systematic toxicity evaluations, and controlled clinical investigations to fully harness purpurin’s therapeutic promise.

## Data Availability

Not applicable.
